# Book Reviews

**Published:** 1830-02

**Authors:** 


### CRITICAL ANALYSES.

Quæ laudanda forent, et quæ culpanda, vicissim

Illa, prius, cretâ; mox hæc, carbone, *notamus*.—Persius.

*A Treatise on Poisons, in relation to Medical Jurisprudence, Physiology, and the Practice of Physic.* By R. Christison, M.D. Professor of Medical Jurisprudence and Police in the University of Edinburgh, &c.—8vo. pp. 698. Edinburgh, 1829.

*Die Lehre von den Giften, in medizinischer, gerichtlicher, und polizeylicher Hinsicht.* Von Dr. K. F. Marx, Professor der Heilkunde an der Universitaet Goettingen. 1er band, 1te abtheilung. Göttingen, 1827.—*A Treatise on Toxicology, in relation to Medicine, Jurisprudence, and Police.* By Dr. K. F. Marx, &c. Vol. I. Part 1. —8vo. pp. 270. Göttingen, 1827.

*An Essay on the Operation of Poisonous Agents upon the Living Body.* By John Morgan, F.L.S. Surgeon to Guy's Hospital: and Thomas Addison, M.D. Assistant Physician to Guy's Hospital.—8vo. pp. 91. London, 1829.

The three works, the titles of which we have prefixed to this article, are important contributions to our toxicological knowledge. The first, which is a complete systematic treatise, is throughout worthy of the author's previous reputation. Comprising a far more extended literature of the subject than any previous toxicological work can pretend to, it contains, at the same time, more ample details of the effects of poison on man, derived from the author's medico-legal experience; for Professor Christison has probably been consulted in a greater number of cases of poisoning, subjected to juridical investigation, than any other British practitioner. The Experimental Essay on Poisoning by Oxalic Acid, published conjointly by Dr. Coindet, of Geneva, and himself; and the elaborate and masterly Essay on Arsenic, contained in vol. ii. of the Transactions of the Medico-Chirurgical Society of Edinburgh, had placed the Professor's talents for toxicological investigation in a very favorable point of view: and the perusal of the present work will not fail to convince all impartial persons, that the same acuteness of judgment, accurate chemical knowledge, and extensive reading, which the author formerly displayed on isolated branches of toxicology, have been brought, in this treatise, to the elucidation of the science in general.

The original chemical details contained in this treatise are particularly valuable, and cannot fail to be most acceptable to practitioners. The author has evinced a thorough practical acquaintance with the methods of detecting *minute* quantities of poisons in *complex* fluids, and has simplified and perfected the methods of analysis previously suggested by ORFILA and others. The processes he has related are delicate, conclusive, and easily managed. Some are novel, and preferable to any previously proposed; and there is scarcely any, the accuracy of which he has not ascertained by frequent trials, under most difficult circumstances.

The second work is by Professor MARX, of Göttingen, the erudite author of the "Origines Contagii." It is as yet incomplete, the portion of it before us being only the first part of the first volume. It is distinguished by the general peculiarity of the German school, that is, by a profound acquaintance with the whole literature of the subject, good and bad; an historical view of which is presented to the reader, without that critical sifting of the chaff from the grain, which by the authors of other countries would be deemed requisite.

This work is intended by Professor Marx to comprehend a view of toxicology in all its relations. As a preparation for it, he remarks, that he has been for many years occupied in considering the subject, and in observing cases in medical practice. He has undertaken a series of experiments on various animals, for the elucidation of the more interesting and complex subjects of toxicological inquiry. He proposes to investigate the *modus operandi* of poisons; the causes of the varieties in their action; to ascertain the medium, that is, the systems or organs, through which poisoning takes place; what are their characteristic symptoms; what effects are produced by them in the body, as ascertainable by post-mortem examination; and the mode of cure, whenever that is practicable. This investigation, with the completion of the general historical details, will occupy the first volume. In the second, poisons will be treated of specially: and under the several poisons, it is proposed, after having given a view of the literature of each, to ascertain its *modus operandi* in man, as well as in animals; the symptoms which it produces; the causes of death, and the kind of death; the post-mortem appearances in man and animals; the mode of treatment; their use as an article of the materia medica, under which head the dose in which it is poisonous will be indicated. The foreign names and most important synonyms will be given, chiefly with a view to illustrate the writings of the ancients. The author has extended his literary inquiries into writings devoted exclusively to criminal jurisprudence, and into the voluminous "Acta Sanctorum;" and from these sources he will derive many curious and some useful facts. A work so comprehensive, executed by a man of talent, judgment, and learning, cannot fail, when completed, to be acceptable to the profession.

The essay of Messrs. MORGAN and ADDISON has been written under a strong impression of the importance of the object of their inquiry; which appears to them to be not merely a question as to the medium by which poisons opèrate on the animal economy, but also as to the mode in which all morbid phenomena are produced in the system; in short, to involve the elucidation of morbid phenomena produced by local agents, of every description, upon the living body. It is designed, then, to illustrate the theory of medicine. Their essay is divided into two parts: a refutation of the theories of former experimentalists, and an experimental attempt to establish their own.

Of the importance of toxicology as a science, it is almost superfluous to offer an observation. Independent of the absolute necessity of its cultivation in relation to medical jurisprudence, it has unquestionably supplied the groundwork for an improved theory of the materia medica. The *modus operandi* of remedial agents was very imperfectly known prior to the modern investigations of toxicological experimentalists; and a proof of this assertion may be gathered from a view of the opinions contained in the works of Cullen, and even Murray. The general fact that agents, however applied, have the same kind of operation on the living body, was formerly unknown; and the study of their physiological actions has much contributed to illustrate their therapeutic operation. "The study of toxicology," says Dr. Christison, "has led to the rejection from the practice of medicine of a host of popular remedies, the offspring of empiricism, which were either totally useless or positively prejudicial."

The object of the science of toxicology is fourfold: it supplies antidotes for the various poisons; it furnishes the physiologist with valuable instruments of research in his investigations into the laws of the animal economy; it aids the physician in his inquiries as to the action of many energetic drugs; and it collects from the numerous branches of medical knowledge, as well as from collateral sciences, the materials of the most important department of medical jurisprudence.

Dr. Christison considers that toxicology has been more successfully cultivated than any other branch of medical jurisprudence, and chiefly because the opinions of medical men have more influence than in any other variety of judicial proceeding. In cases of poisoning, many causes combine to concentrate the weighty part of the proof in the medical evidence. The proof of the fact, or of death having been occasioned in the manner alleged, can very seldom be drawn, as in other cases of homicide, from general evidence, or from any thing else than medical testimony. This evidence is the more important, that the proof of poisoning also commonly infers proof of the intent: for on such trials it is impossible, as in other trials, to entertain the question whether death was the consequence of deliberate purpose, or of sudden fury, or of an act of self-defence.

After a luminous exposition of the objects and importance of toxicology, Dr. Christison proceeds to develope the views which have influenced him in the composition of his work. Having alluded to his own frequent medico-legal engagements, as some warranty for executing the present treatise, he comments on the omissions of practical points in existing works, possessing in other respects high reputation and scientific excellence. Some inquiries have not been noticed at all, and others very cursorily handled; and these defects he ascribes to the attention having been too exclusively turned to the means by which particular poisons may be proved to have been the cause of death; whereas, the questions which actually occur in medico-legal practice are much more diversified. Dr. Christison has endeavoured to supply these defects, in his chapters on General Poisoning and on the Diagnosis between the Effects of the Irritant and Narcotic Poisons and the Effects of Natural Disease.

Medical testimony is required, first, before the coroner in England or the sheriff in Scotland, as a sort of preliminary investigation; and, secondly, before the higher criminal courts. In the former, the question to be decided is, whether there is a certainty, probability, or possibility of poisoning, in a general sense. In the latter, the prisoner is charged with administering a particular poison. But in some instances the evidence of the particular poison is merely presumptive, and that presumption not strong, so that the charge is substantially one of poisoning in a general sense; and convictions have been obtained in some trials in Scotland, where no satisfactory proof existed what poison had been given.* Dr. Christison dissents from the opinion expressed by almost all continental medical jurists, who affirm the insufficiency of the proof derived from the evidence of general poisoning alone. "It is very likely," he admits, "that the proof of general poisoning from medical evidence alone, can never amount to more than a strong probability. But the medical probability may be so high that, in conjunction with other circumstances of general evidence, no rational being can entertain a doubt that poisoning has been perpetrated."

The chapter on General Poisoning contains valuable practical remarks, which will be sought in vain in Orfila, or other writers on toxicology. (Vide Orfila, *Toxicologie générale*, ii. 605.) When the charge made is of poisoning by some particular poison, it is investigated by chemical analysis, by the morbid appearances found in the dead body, by the symptoms during life, and by the effects of the suspected poison on animals. Dr. Christison has thus arranged his investigations of particular poisons.

In treating of the symptoms observed in man, Dr. C. has not followed the example of Orfila. The latter has transcribed a list of complete cases: the former has given a general account of the effects of each poison. We think a few *selected* cases preferable to either plan, as conveying instruction in a more natural and striking form. The operation of many poisons is various in different individuals, but the differences are referrible to a few varieties; and the narration of an example, as it occurred in practice, of each variety of the mode of action of each poison, would convey clearer and more impressive instruction than any artificial grouping of the symptoms, however skilfully executed. Professor Orfila's mode has unnecessarily expanded his work: Professor Christison's presents an assemblage of symptoms never occurring in any one single instance. Dr. Christison has added valuable information on the shortest and longest known intervals within which poisons begin to operate, and on the longest and shortest known periods within which they prove fatal. This kind of information is not to be met with in any previous

* Vide Christison, pp. 42, 64, and 71.

systematic treatise on Toxicology. He has discussed also the *treatment* to be pursued in the principal varieties of poisoning. We proceed to analyse the work more closely.

It consists of two parts, to which is added a brief appendix. The first part treats of general poisoning, and is divided into three chapters, of which the first treats of the physiological action of poisons; the second, is "of the evidence of general poisoning," as ascertainable by symptoms, by morbid appearances, by chemical analysis, and by moral proof; and the third, treats briefly of "imaginary, pretended, and imputed poisoning."

Part the second includes the individual poisons, and consists of thirty-eight chapters. Chapter the first is on the Classification of Poisons, which our author, adopting physiological action as the most convenient basis of arrangement, has divided into irritant, narcotic, and narcotico-acrid, properly rejecting the class of septic, which Orfila had admitted. The same distribution has been adopted by Professor Bernt,* and there can be no doubt of its superior accuracy, whether practical utility or scientific accuracy be considered. In detail, it may be open to some objection; but it is the only arrangement which connects the poison intimately with the symptoms and pathology.

Chapter the second treats of Irritant Poisons generally, and contrasts the symptoms and morbid appearances of natural disease with those produced by this class of poisons. Then follow the most important individual poisons of the class, and these occupy the work down to the twenty-fourth chapter. The twenty-fourth chapter is on Narcotic Poisons, and contrasts the effects of them with the effects of natural disease. This class occupies five chapters. Chapter twenty-ninth takes up the subject of the Narcotico-acrid Poisons, and they are continued through the nine succeeding chapters.

To attempt a detailed analysis of a work so comprehensive, would be inconsistent with the limits which can be assigned to it in our Journal; nor, indeed, would it be very practicable to give a clear and instructive abstract of the whole treatise, which will be readily understood when we state that the skilful condensation of the materials collected

* Venena corrosiva; stupefacientia vel narcotica; et corrosivo-stupefacientia." (Bernt's "Systematisches Handbuch der gerichtlich. Arzneikunde," 199. Dr. Marx has divided them into irritant or corrosive, narcotic, narcotico-acrid, and "austrocknende," desiccating, we presume, though what can be comprehended under this class we cannot divine, and his work has not proceeded far enough to furnish any illustrations.

by the authors' extensive research, is one prominent excellence of the work. Hence we shall confine our remarks to a few leading subjects.

In the chapter on the Mode of Action of Poisons, Dr. Christison divides their operation into *local* and *remote*. The local effects are of three kinds. They *corrode* or chemically decompose the part to which they are applied; or, without immediately destroying its organization, they *inflame* or *irritate* it; or, without producing either corrosion, inflammation, or irritation, they make a peculiar impression on the sentient extremities of the nerves, unaccompanied by any visible change of structure. Many of the irritants (as arsenic, for example,) are, in common speech, called corrosives, but they do not occasion chemical decomposition: it is by means of inflammation, and its effects alone, that any breach of continuity is produced.

Dr. Christison adopts the term *remote*, in preference to the more common phrase, *general* action, because the latter implies an action on the general system, or whole body. Such an affection of the entire system is, however, rare: it is one or more of the important organs only which suffer from the indirect action. This remote action of poisons is well illustrated by the effects of oxalic acid. Concentrated oxalic acid acts as a corrosive by destroying the gelatine of the animal textures, yet it never kills by destroying the function of the stomach. It has proved fatal to man in ten minutes, and to a dog in three. Nor does it always induce, when swallowed, symptoms of an injury of the stomach; for death is often preceded by tetanus or apoplexy, or mortal faintness. Hence death commences, so to speak, in the spinal marrow, brain, or heart; and, in fact, death is most rapid under circumstances in which the stomach is least injured, namely, when the acid is considerably diluted.

Concerning the channel by which the remote influence of poisons is excited, some fluctuation of opinion has taken place. Until very recently, the opinions of modern toxicologists has been very much guided by the experiments of Magendie; and scarcely has any one hesitated to assent to the opinion that poisons are transmitted by venous absorption into the circulation, and thence conveyed to the brain or other organ remotely affected. Lately, however, this hypothesis has been controverted by Messrs. Morgan and Addison, in the essay to which we have already alluded. The theory which these gentlemen believe to be most consistent with sound reasoning, and also fairly deduced from experiment, is contained in the following proposition: "That all poisonous agents produce their specific effects upon the brain and general system through the sentient extremities of nerves, and through the sentient extremities of nerves only; and that, when introduced into the current of the circulation in any way, their effects result from the impression made upon the sensible structure of the blood-vessels, and not from their direct application to the brain itself." (Essay, &c. p. 60.) And to this theory Dr. Christison has given the sanction of his approbation.

Messrs. Morgan and Addison have arrived at their conclusions, partly from certain assumptions and reasonings, and partly from experiment. They assume that such is the rapidity with which death takes place from some poisons, that the absorption of the poison is impossible; and hence, as it is absurd to suppose that nature employs two modes of effecting the same purpose in the animal economy, absorption never can be the direct medium of transmission; that the phenomena of sensation, and some of the morbid effects of mechanical injuries, analogous with the effects of some poisons, occur without the absorption and subsequent application of any material agent to the brain or other organs affected; and, finally, contending for a common medium through which all morbific agents produce their remote or general effects, they reject the notion that absorption supplies that medium, in the sense contended for by Brodie, Magendie, &c.

After an attentive consideration of their reasoning and facts, and a comparison of them with others already before the public, we do not hesitate to express our dissent from their hypothesis. Their analogical reasoning appears to us inconclusive, their experiments exceptionable, and the inferences deduced from them untenable, or liable to considerable doubts.

That absorption is the direct medium by which the influence of poisons is conveyed to the brain or other organs remotely affected, is the opinion which they have controverted. This opinion, in our judgment, is full as plausible as that now proposed by our authors; either of them, however, demands further experiments to confirm or refute it.

We beg to make, in this place, a few remarks on the phenomena of sensation, and on the sudden mortal effects of some mechanical injuries, in relation to the present subject.

Sensation, an ultimate fact in physiology, is a function performed by a special apparatus, that is, by the nerves of special sense, and by the ramifications of the posterior branches of those which Mr. C. Bell has denominated double nerves; and, however it may be employed as an illustration, has but one analogy with the effects of poisons, namely, rapidity of transmission. Now, it is experimentally demonstrated of many poisons, that when they are applied either to the nerves of sensation or to those subservient to voluntary motion, their remote effects are not produced. Neither is the integrity of these nerves in a limb to which poison is applied at all requisite for the full and rapid production of its remote effects. Hence it appears that whatever analogy may be supposed to exist between the sensations and the effects of poisons, the medium through which their influence is transmitted is not the same: consequently, that there must be some apparatus connected with the brain and spinal marrow, &c. independent of the nerves of sensation and volition, through the medium of which the phenomena of poisoning may be sometimes excited with a rapidity *like* that of sensation.

We willingly concede to Messrs. Morgan and Addison, that the *medium* through which poisons, commonly so called, and many other (not all, however,) morbific agents, produce their remote or general effects is one and the same; and that no ground of distinction can be drawn as to the nature of morbific agents from the interval of time which elapses between the application of the cause and the production of its effect; and we, further, see no reason to controvert their position, that we find an *analogy* in the nature of the effect, as well as in the period of their occurrence between the sensible consequences of morbific agents and mechanical injuries, as in tetanus, and the sudden mortal effects of gunshot wounds, &c. But we think it is obvious that they have deceived themselves in confounding the operation of causes which operate through sensation* or mental emotion (and exciting either morbid or fatal sympathies) with others which have no such source of operation, and that, in fact, they have too much limited their views into the causes of disease.

Absorption, then, being denied to be the medium through which the remote effects of poisons are produced, and nervous transmission its alleged medium, it remains to be asked to what part of the nervous system this function is attributed? We have seen that the nerves of sensation and

* Vide Dr. Alison's paper on Sympathy in vol. ii. of Med.-Chirurg. Trans. of Edinburgh; and Travers on Constitutional Irritation.

volition are excluded, and the authors have not mentioned the sympathetic nerve and ganglionic system. We presume, however, that this must have been in their contemplation; and, if such was their opinion, it was susceptible of some illustration by direct experiment. We cannot, however, avoid expressing our expectation that the result of such experiments, if they had been instituted, would have been unfavorable to the views of Messrs. Morgan and Addison. (Vide Milligan's Magendie, p. 97.)

In those slight or severe gunshot wounds and other mechanical injuries, from which sudden mortal effects are said to have occurred, without there being any perceptible sign of violence, it can never be satisfactorily proved that mortal faintness was not produced by mental emotion. Mental emotion is known to be capable of producing such effects; and it is probable that, when such cause is absent, these mortal effects are referrible to sudden and painful sensations. To tetanus from mechanical injury, as an illustration of the theory of Messrs. Morgan and Addison, we decidedly object. Authentic histories of tetanus, thus occasioned, do not mention its occurrence till after the lapse of some days, and what processes may have been going on during that time is unknown. It may hereafter turn out that this illustration is decidedly unfavorable to their mode of explanation.

Poisons, it appears, then, may produce their effects with a rapidity somewhat like the phenomena of sensation, and sensations may produce morbid phenomena in the general system like the effects of poisons; but sensations are transmitted by an apparatus through which the remote effects of poisons are not produced: neither is the integrity of sensation essential to the action of some poisons. Sensations and mental emotion can produce fatal effects; but if death from either commence, as is probable, in the cerebro-spinal system, indirectly affecting the heart, &c., the medium through which the former operate is familiar; and with regard to the latter, it may be safely affirmed to constitute no formidable opposition to the theory of Magendie, &c. The notion that the rapid effects of some poisons cannot, by possibility, be attributed to venous absorption, is obviously gratuitous; and no attempt has been made by Messrs. Morgan and Addison clearly to point out by what system of nerves they conceive them to be transmitted. Nevertheless, we are far from thinking that there has been any experimental demonstration of the theory that poisons are necessarily conveyed to the parts affected in their remote action; but there are some strong probabilities in its favor.

It is well ascertained, for example, that the blood is impregnated by the poison. It is known also that the rate of venous absorption influences the rapidity and degree of the effect of poisons; and so far as any direct experiments have been made to ascertain in how short a time poisons can traverse the system, the result is favorable to the theory of Magendie; and, finally, experiment demonstrates that the remote effects of poisons are produced independently of that system of nerves which has the closest connexion with the cerebro-spinal system. We proceed to give the proofs in support of these probabilities.

A case of poisoning by oxalic acid was communicated to Dr. Christison by Dr. Arrowsmith, which proved fatal in thirteen hours. Six hours after the poison was swallowed, some leeches were applied to the region of the stomach, and were almost immediately poisoned. "They were healthy," says Dr. Arrowsmith, "small, and fastened immediately. On looking at them in a few minutes, I remarked that they did not seem to fill, and, on touching one, it felt hard, and immediately fell off, motionless and dead. The others were all in the same state. They had all bitten, and the marks were conspicuous, but they had drawn scarcely any blood."* This is an isolated fact, but it appears to be sufficiently well attested. Since Dr. Arrowsmith made this observation, M. Vernière has pointed out the extreme susceptibility of the medicinal leech to the effects of poisons.

After a *fatal* dose of extract of nux vomica had been thrust into the paw of a dog, M. Vernière applied a tight ligature around the limb; warm water was then very slowly injected into the jugular vein to as great an extent as the animal could safely bear, and the ligature was then removed. Half an hour was allowed to elapse, which in ordinary circumstances would have been much more than sufficient to enable the poison to act, but the animal remained totally unaffected. The ligature was next replaced, but so as to obstruct the venous circulation only just as in performing the operation of venesection; the principal vein of the limb was then opened immediately below the ligature, and the blood which flowed was collected. This blood, cautiously injected into the vein of another dog, caused violent tetanus and almost instant death. The inoculated dog remained unaffected. (Journal des Progrès des Sciences, tom. iii. 1827.)

The "vaunted" experiment of M. Magendie (as our authors term it) has unnecessarily encountered much of

* Christison, p. 146.

their criticism. In this well-known experiment, M. Magendie separated the limb from all connexion with the body, except by the femoral artery and vein. He then divided them, and connected the divided ends by quills, so that the blood which returned from the limb necessarily passed through the quill, and the quill only. Poison introduced into the paw destroyed this animal as speedily as though no mutilation had taken place. M. Magendie considered this experiment to be a demonstration of venous absorption.

There are other experiments, however, of M. Magendie quite as worthy of our authors' attention. We beg to quote some of them, as they illustrate the above experiment of M. Vernière.

"After having injected almost two pints of water into the veins of a dog of ordinary size, I introduced into his pleura a small dose of a substance, with whose effects I was familiar. I was surprised to see the effects only take place several minutes after the period at which they usually show themselves.

"In another experiment wherein I had introduced as much water (about four pints) as the animal could support without ceasing to live, the effects did not show themselves at all. Absorption had probably been prevented. After waiting almost half an hour for effects which only require about two minutes to display themselves, I reasoned as follows: if the distention of the blood-vessels be, in this case, the cause of the absence of absorption, then, distention ceasing, absorption ought to take place. Immediately I took a large bleeding from the jugular vein of the animal submitted to experiment, and I perceived, with singular pleasure, the effects proceed to manifest themselves as the blood flowed.

"An animal was bled, and lost half a pound of blood: effects (that is, of a poisonous agent introduced into the pleura,) which should not have happened before the end of the second minute, showed themselves distinctly before the thirtieth second." (Journal de Physiologie, tom. i.)

Now, although it is plain that the preceding observation and experiments leave the question of the transmission of the effects of poisons by "the sensible structure of the blood-vessels" undetermined, they establish two facts, namely, that the blood is fully impregnated by the poison, and that the rapidity and the degree of effect of some poisons, if not of all, is regulated by the rate of absorption; and they pointedly refute the following assertion of Messrs.

Morgan and Addison, (p. 50,) that "M. Magendie has left the question relative to the *necessity for venous absorption* and cerebral contact, as connected with the operation of poisonous agents, in precisely the same state as he found it."

Mr. Brodie admitted (incautiously, we think,) that the rapid effects of some poisons could not be transmitted through the medium of absorption, which medium he had previously contended for where the effects were developed more slowly. Hence he assumed a double medium of communication with the brain in the operation of poisons. Our authors assume this probable mistake of Mr. Brodie (the rejection of absorption as the medium by which the most rapid effects of poisons are conveyed,) as an indisputable truth, and comment rather sharply upon the inconsistency of his philosophical creed. We agree with them in thinking that "it is contrary to all fair analogy to suppose that any variety observed in the effect of a local agent can essentially depend on the medium by which it is carried into the system;" and we think they would have no objection to add, that poisons, in a concentrated state, must act remotely through the same channel as the same poisons in a milder form, and hence that the more rapid effect produced by a concentrated poison is in itself no argument for any particular channel of communication; and, as in experiments where the effects are more slowly developed, the phenomena are more easily observed, the milder mode of producing such phenomena appears the most likely, we think, to enable us to detect this disputed channel.

This inference of Mr. Brodie, so eagerly seized on by his "opponents," is supported by no direct evidence. Dr. Christison has collected in his work facts to "show the shortest and longest intervals within which poisons begin to operate; and the shortest and longest period within which they prove fatal." But direct experiments to prove in how short a time poisons may traverse the different organs of the body have been very scantily supplied. The following, from Tiedemann's Physiological Journal, are interesting:

"The time," says Professor Hering, "which a solution of ferro-prussiate of potass, injected into the one jugular vein, requires to reach that of the opposite side was, in various experiments, from twenty to twenty-five or thirty seconds; to reach the external thoracic vein of the other side, from twenty-three to thirty seconds; the vena saphena major, twenty seconds; the masseter artery, from fifteen to thirty seconds; the opposite maxillary artery from ten to fifteen seconds," &c. (Zeitschrift für Physiologie, band iii. s. 85.)

The concentrated state of poisons obviously augments the rapidity of their effects; and we may observe, *en passant*, that the substance experimented with by Professor Hering is but little poisonous. In addition to the state of concentration, we beg to suggest that the *physical properties* of a poison may also influence the rapidity of its effect. Prussic acid is the poison by which the most rapid effects have been produced. Now, prussic acid is very volatile; it boils at 80° F.; and is it unreasonable to suppose that, when exposed to the heat of the blood, as in dropping it into the jugular vein, its very volatile nature in the concentrated state may so facilitate its transmission by the blood to remote parts, so as to render it unnecessary to call in the aid of any other channel? In cold-blooded animals its effects are less sudden; and when the vessels of any part are tied before the part is touched with the prussic acid, its action is prevented; but the previous division of the nerves of a part has no such effect.

We are not aware that any more direct or conclusive evidence exists to prove that poisons are actually conveyed to the parts remotely affected; but we think the preceding facts and observations render such an opinion probable, and that they weaken the objections brought against it from the great rapidity of the remote action of some concentrated or gaseous poisons.

It remains for us to show in what particulars the experiments of Messrs. Morgan and Addison are open to objection, and their inferences doubtful; and we shall conclude with explaining, that if the result of their experiments had been the reverse of what they were, the objections advanced by those gentlemen to explain the fallacies of all preceding experiments are equally applicable to their own. Hence a true *experimentum crucis* is as yet a desideratum.

Messrs. Morgan and Addison relate two experiments to prove the susceptibility of the internal coats of the veins to poisons.

The circulation in the jugular vein of a dog was interrupted. The vein was divided, and afterwards connected by means of a brass cylinder containing some woorara. In forty-five seconds, all power over the voluntary muscles ceased; in two minutes, convulsions and respiration had entirely ceased. This result is altogether usual.

But, secondly, a dog of the same size had the circulation temporarily arrested in the jugular vein, and afterwards a quill containing woorara was introduced within the vein, through a small opening; the impediment to the circulation *above* the quill was removed, so that the blood came in contact with the poison; the *lower* ligature was retained, so that the direct communication with the heart was interrupted. This animal dropped in convulsions in 108 seconds, and expired in three minutes and a quarter.

We are at a loss to conceive how these experiments can be supposed to prove an action through the nervous fibrils of the vein, rather than by the round of the circulation.

Further to show the instrumentality of the *sensible* structure of the blood-vessels, poison (woorara) was introduced into the carotid artery in two animals, and into the femoral artery of another: but although, say our authors, the poison was directly conveyed to the brain, the convulsions and death happened no earlier than in the previous experiments, when the poison was introduced into the veins. But on this experiment we would remark, that the period in which death is produced varies in different poisons, and what is the shortest time within which woorara proves fatal has not been particularly ascertained. It may turn out to be the time mentioned in these experiments.

But, to prove that the poison was not dissolved in the blood, (Essay, p. 84,) and thus conveyed to the brain, a connexion and circulation through the carotid of one dog with the carotid artery of another dog was established, so that the blood from the heart of one dog was conveyed to the brain of the other dog. The "poison of nux vomica" was then introduced into the back of the animal whose blood was transmitted to the brain of the opposite animal. The inoculated animal was affected in the usual manner, and so continued for fourteen minutes, during which period the circulation was maintained, but the other animal did not furnish the slightest indications of its influence. (P. 82.)

To this experiment we oppose the following considerations: The diameter of one carotid artery, in its healthy state, is little more than one fourth of the whole caliber of the vessels carrying blood directly to the brain: consequently, the dog not inoculated was subjected to the influence of one fourth only of the quantity of the poison which was conveyed to the *brain alone* of the inoculated animal. A great proportion of the poison absorbed and dissolved in the blood must, however, have been distributed by the blood to other parts also of the inoculated animal, and particularly (be it remarked) to the medulla spinalis. Now, so far as a satisfactory pathology of tetanus and tetanic affections has been made out, it is highly probable that the seat of such pathologic state is in the medulla spinalis; and, from experiment, it seems no less probable that the action of strychnia is almost peculiar to the same part of the cerebro-spinal system. For proofs of these opinions we refer to the works of Ollivier, Abercrombie, of Bellingeri, C. Bell, and Magendie and Delille. It may be worth while, indeed, to quote two experiments performed by the two last-mentioned gentlemen, with the Upas tieuté, which Pelletier and Caventou inform us contains strychnia for its active principle.

After relating an experiment in which, the spinal marrow having been divided at the occiput, the tetanic convulsions followed the employment of the poison as usual, they performed the following: "Eight drops of upas, diluted with water, were injected into the pleura of a strong dog: at the same instant a piece of whalebone was forced down the whole length of the vertebral canal; the whole of the spinal marrow followed the whalebone when it was withdrawn from the vertebral canal. *Ten minutes* after the destruction of the spinal marrow, the circulation continued, and there was *no convulsion*.

"In another experiment, the same quantity of upas was injected into the cavity of the peritoneum of a dog: the moment the 'tetanus' manifested itself, a piece of whalebone was forced down the vertebral canal, commencing with the first cervical vertebra: the tetanus ceased in the fore paws when the whalebone reached the dorsal region; it continued, on the contrary, in the posterior extremities, which ceased to be convulsed when the piece of whalebone reached the caudal extremity of the vertebral canal." (Orfila, Toxicologie générale, ii. 369.)

Now, the preceding considerations, and the facts connected with them, serve to explain, first, how very different were the quantities of poison administered to the two dogs; and, secondly, how much more considerable was the quantity directed towards the spinal marrow in the inoculated dog than in the other. Nor is it altogether groundless to presume (from a remark which occurs in the following experiment,) that the connexion of the two arteries, by means of an inelastic tube, would retard the flow of blood in the uninoculated, and thus further diminish the quantity of the poison sent to the brain. The therapeutic experience of our authors must, of course, have convinced them that nux vomica is not poisonous in every dose.

Previously the authors had not thought it worth while to dispute (p. 79,) whether the poison does circulate with the blood; but, from this experiment, they appear to have concluded (how erroneously our readers must perceive) that it does not mingle with the blood.

An experiment, apparently more conclusive than the preceding, or indeed than a modification of it subsequently related in their work, is described at p. 85. In this experiment, the division and reconnexion of the jugular veins in each dog was effected in the manner formerly alluded to, "so that the venous blood from the head of one dog passed into the heart of the other. The animal contributing blood to the other was then inoculated on the side of the face with nux vomica, and in the usual time exhibited the usual symptoms: these continued without intermission during the space of *seven minutes* after the animal was first affected, the circulation being freely kept up through the artificially connected jugulars; at the end of this period, *the circulation was beginning to become impeded by the formation of a small coagulum in the tube.*" The uninoculated dog never showed the slightest symptoms of being poisoned.

It is a sufficient objection to this experiment, to say that the *whole* of the poison inserted into the face could not, under any such circumstances, be transmitted by one jugular vein; but we have evidence enough that the circulation, so far from having been freely kept up, was much impeded, for, in the short space of seven minutes, a coagulum had formed within the tube, which, if the experiment had been continued, would probably have arrested the circulation through the vein entirely. It is inconsistent to suppose there would have been any *tendency* to the formation of a coagulum, if the natural velocity of the circulation had been maintained; and we conceive there will be but little hesitation in admitting that, if *any* impediment exist to the free transmission of the blood in one quarter, it is immediately diverted to another. In this experiment, therefore, (certainly the best performed by our authors,) it is exceedingly uncertain what dose of the poison passed into the circulation of the uninoculated dog. M. Vernière's experiment demonstrates that the blood is poisoned by nux vomica, and hence we must infer that, in this experiment, the quantity conveyed by the jugular vein in this uninoculated dog was not a poisonous dose.

We think the warmest partizans of the theory of Messrs. Morgan and Addison will allow that the preceding facts and observations satisfactorily explain the inconclusiveness of their reasonings and experiments; and that, if their theory be the true one, it must be proved to be so by further investigations; and we beg to ask, if the result in the two last experiments had been just the reverse of what it was, that is, if the uninoculated animals had been poisoned by the blood of the inoculated animals, whether it would not have been easy for them to say, "this is no proof against our theory: the poison came in contact with the 'nerves of the inner coat of the blood-vessel' (p. 79,) before it reached the brain, and in this way was its influence transmitted." Nor could that assertion be refuted. Hence their "vaunted" experiments do not offer to us an *experimentum crucis*.*

Now, we venture to presume that the results of analogous experiments will be found the reverse of those which they have obtained. But the experiments must be modified. We beg to suggest that concentrated prussic acid, strychnia, &c. in large doses, be tried; and, as of course the inoculated animal will, under such circumstances, be speedily destroyed, respiration should be maintained artificially. If the *blood be strongly impregnated with the poison, and circulate freely*, we confidently anticipate that the uninoculated animal will experience its fatal influence. The theory of Messrs. Morgan and Addison will then repose on their inconclusive reasonings for its only support, and the probability of the views of Magendie, Barry, &c. will be increased. But an *experimentum crucis* will still be a desideratum.

It is scarcely necessary to add, that the treatment of poisoned wounds recommended by Dr. Barry remains entirely unaffected by the experiments and speculations of our authors; whilst we regret to say that their criticisms of Dr. Barry are not particularly candid. Indeed, the supercilious and oracular tone which pervades the essay is rather misplaced in a first attempt at physiological speculation.

With these remarks we conclude our notice of the essay of Messrs. Morgan and Addison, and proceed to consider Dr. Christison's chapter "on the Evidence of General Poisoning."

The investigation of the evidence of general poisoning is purely medico-legal. It comprehends an account of the various kinds of evidence by which the medical jurist is enabled to pronounce whether poisoning, in a general

* We recommend to our authors' consideration the experiments of Magendie on the vena porta, into which he injected substances of a poisonous nature. By what nervous fibrils is that vein supplied?

sense (that is, without reference to a particular poison,) is impossible, improbable, possible, probable, or certain. It likewise comprises an appreciation of the circumstances which usually lead the unprofessional, as well as the professional, to infer, correctly or erroneously, a suspicion of such poisoning. (Christison, p. 30.)

The evidence by which the medical jurist is enabled to pronounce on the existence or non-existence of poisoning in general, and to determine the subordinate questions that relate to it, is derived from five sources: 1st, symptoms during life; 2, appearances in the dead body; 3, chemical analysis; 4, experiments and observations on animals; and, 5, certain moral circumstances, which are either inseparably interwoven with the medical proof, or cannot be accurately appreciated without medical knowledge.

### I. *Of the evidence from symptoms.*

So lately as during the latter part of the 18th century, opinions were grounded almost exclusively on the symptoms. About that time the infallibility of such evidence began to be doubted, and it is now laid down by every esteemed writer on medical jurisprudence, that these symptoms, however exquisitely developed, can never justify an opinion in favor of more than high probability. Dr. Christison dissents in some measure from this doctrine; and in laying it down, medical jurists, he says, appear to him to have confounded actual symptoms with their general characteristics. He thinks this doctrine correct as far as regards the *general characteristics* merely of the symptoms, but, if applied to the actual symptoms produced by particular poisons, in all cases whatever of their action, it is liable to several exceptions. For example, if, after something has been taken which tasted acrid, and caused a sense of heat, pricking, or tightness in the throat, the characteristic signs of poisoning with the irritants make their appearance in the usual time, and are soon after accompanied or followed by true mercurial salivation, it may be safely inferred that some soluble compound of mercury has been taken. If, immediately after swallowing a liquid which causes a sense of burning in the throat, gullet, and stomach, violent vomiting ensues, particularly if the vomited matter is mixed with blood; if the mouth become white or yellow, and stripped of its lining membrane; and the cheeks, neck, and neighbouring parts show vesications, or white, or subsequently yellow or brown excoriated spots; if the clothes show red spots, and are disintegrated there, Dr. Christison thinks the inference inevitable that nitric or sulphuric acid has been taken; and he is supported in his notion of the sufficiency of such proof by Dr. Mertzdorff, late medical inspector of Berlin.

If a person, immediately after swallowing a solution of a crystalline salt, which tasted purely and strongly acid, is attacked with a sense of burning in the throat, and then in the stomach, and vomiting, particularly of bloody matter; imperceptible pulse and excessive languor; and dies in half an hour, or, still more, in twenty, fifteen, or ten minutes, Dr. C. knows of no fallacy which can interfere with the conclusion that oxalic acid was the cause of death. No parallel disease begins so abruptly and terminates so soon, and no other crystalline salt has the same effects. Should a person be taken several times ill with symptoms of general inflammation of the mucous membranes, and each time after partaking of a suspected article of food and drink, the proof of the administration of arsenic would be very strong indeed, and it would be unimpeachable if at length a nervous affection succeeded at the usual period. Or, above all, suppose several persons, who have partaken of the same dish, are seized about the same time with nearly the same symptoms of irritation of the mucous membranes, the proof of general poisoning would then be unequivocal.

To return from these exceptions. The chief characteristics usually ascribed to the symptoms of poisoning, considered generally, are, that they commence suddenly, and prove rapidly fatal; that they increase steadily; that they are uniform in nature throughout their course; that they begin soon after a meal,* and that they appear while the person is in a state of perfect health. These are general facts, but all liable to exception. In instances of slow criminal poisoning, and of poisoning whilst a person is labouring under natural disease, they do not hold good. Cases of the latter description are generally very embarrassing; for if, instead of medicine, a poison be administered, the symptoms occasioned by which resemble the natural disease, suspicion may not arise till it is too late to collect evidence.

Now, although the characters common to the symptoms of general poisoning are by no means universally applicable, yet, considering the little knowledge possessed by the vulgar of the action of poisons, and, consequently, the rude nature of their attempts to commit murder by poisoning, the exceptions will not be numerous; and the chief characteristics will often enable the jurist to say that poi-

* The negative evidence on this point is favorable to the person accused, and sometimes decisive against poisoning.

soning was possible, probable, or highly probable; which, when the moral evidence is very strong, may be quite enough to decide the case; and, although they can never enable him to say that poisoning was certain, they will sometimes entitle him to say, on the contrary, that it was impossible; and, when the chemical or moral evidence proves that poison was given, the characters of the symptoms may be necessary to determine whether it was the cause of death.

We think it will be convenient to continue, in this place, the symptomatology of the different classes of poisons, as contrasted with the symptoms of various natural diseases; although, in so doing, we depart widely from the arrangement of Professor Christison.

1. And first of the class of irritant poisons. Dr. Christison, after stating that inflammation of the whole alimentary canal, more or less, is the chief consequence of these poisons, adds that they are accompanied, in almost every instance, with great disturbance of the circulation, quick feeble pulse, excessive prostration of strength, coldness and clammy moisture of the skin; and sometimes symptoms of irritation and inflammation of the windpipe and lungs, and with those of irritation of the urinary organs.

The most important natural diseases whose symptoms have an analogy with the preceding, are distention and rupture of the stomach; rupture of the duodenum; effects of drinking cold water; bilious vomiting and cholera; acute gastritis; inflammation of the intestines; peritonitis; spontaneous perforation of the stomach; melæna and hæmatemesis; colic, iliac passion, and obstructed intestine. (Christison, 87; Orfila, *op. cit.* ii. 606.)

*a.* Death from distention of the stomach may take place either from superinduced apoplexy, &c. or from simple over-distention of the stomach, but a careful post-mortem examination removes all doubts. *b.* Rupture sometimes arises from over-distention, particularly when combined with efforts to vomit, for the cardiac aperture becomes valved, and the contents cannot be discharged by vomiting. Sometimes from distention by gases developed by depraved digestion; and sometimes there is a partial rupture, or laceration of the inner coat only. Rupture may occur without previous distention, and death is sometimes instantaneous. The symptoms of rupture from distention, and of ventricular perforation, are a sense of something giving way in the pit of the stomach, acute pain gradually extending over the whole abdomen, great tenderness and tension, excessive prostration, &c. *c.* Ruptured duodenum is rare, and requires no particular notice. *d.* Drinking cold water when the body is heated sometimes produces sudden death, and not uncommonly instantaneous death. Sometimes it produces apoplexy, and in some instances cholera. Of all diseases, this last is, however, the most embarrassing, on account of its frequency and peculiar symptoms; and some cases of irritant poisoning, says Dr. Christison, cannot be certainly distinguished by their symptoms from cholera, but in some others, where the physician has been able to ascertain the symptoms in detail, the distinction may be drawn. The sense of acridity or burning in the throat never begins in cholera before vomiting; in poisoning, it sometimes does: and Dr. Christison says, that no case is recorded where the vomiting in cholerâ was bloody. And, lastly, cholera in this country is not very often fatal, and, when it is so, death very rarely takes place within three days. Two or three instances are, indeed, recorded of death within twelve hours from cholera in this country; and it should be observed, that cholera generally prevails at a particular season of the year, and in an epidemic form. *f.* Acute gastritis. Dr. Abercrombie has said that he has never seen a case of acute idiopathic gastritis, nor have Dr. Christison's inquiries discovered more than one or two *probable* cases of that description. In this disease, as in cholera, it is important to observe that the sense of burning, if present at all, does not precede vomiting. *g*, *h*. Should enteritis or peritonitis present some analogies with the symptoms of irritant poisons, they are readily distinguished by post-mortem examination. *i.* Spontaneous perforations of the stomach are sometimes accompanied both with symptoms and post-mortem appearances resembling the effects of irritant poisons. This subject is rather obscure, and is highly deserving of further investigation. Spontaneous perforation is of three kinds. One is the last stage of some varieties of scirrhus. This kind of perforation is generally fatal within twenty-four hours. The previous disease is not always indicated by pathognomonic signs. The second variety of perforation takes place by simple ulceration, without previous scirrhus. The third variety is much more singular, and is the result of *ramollissement*. It is not necessarily accompanied by vascularity; the symptoms are very obscure. In addition to the authors mentioned by Dr. Christison and others, some observations on this subject are to be found in the third volume of the Transactions of the Austrian Physicians, by Professor Lenhossek, who, we believe, anticipated the views of Dr. Gairdner; and also in the work of M. Billard on the Diseases of Infants. By Hunter, Allan, Burns, and other British pathologists, this state has been attributed to the solvent power of the gastric juice, and in some instances correctly, but certainly not in all. We refer to the thesis of Laisnè, the observations of Dr. Gairdner, Lenhossek,* and Billard,† for illustrations. *k.* Perforations of the gullet and intestines have not been observed as the results of poisoning. *l, m.* Melæna and hæmatemesis it is scarcely possible to mistake for effects of poisoning, as the pain which accompanies them is seldom acute, and the discharge of blood is generally profuse. *n.* Colic, iliac passion, and obstructed intestine. The abdominal symptoms alone do not seem to distinguish these natural diseases from the effects of poisons: but, in severe cases of poisoning, collateral symptoms will almost always furnish a ground of distinction. The symptoms of these affections are well known. Dr. Christison says he is not aware that stercoraceous vomiting is ever occasioned by poisoning. In colic from mechanical obstruction, the seat where the pain commences, and the obstinate constipation, form grounds of distinction.

2. Of the symptoms of the narcotic class of poisons compared with the symptoms of natural disease.

The symptoms of the narcotic poisons in man, and in the higher order of animals, are giddiness, headach, obscurity or deprivation of sight, stupor or perfect insensibility, palsy of the voluntary muscles, or convulsions of various kinds, and, towards the close, complete coma; and the most important diseases whose symptoms may be confounded with the preceding are apoplexy, epilepsy, inflammation of the brain, hypertrophy of the brain, inflammation of the spinal cord, and syncopal asphyxia.

*a.* Apoplexy has often precursory symptoms. M. Rochoux has said, however, that, of sixty-three cases which came under his notice, nine only had distinct precursory symptoms. It attacks chiefly those advanced in life, although that is not without numerous exceptions. It occurs chiefly amongst the corpulent, and sometimes during a meal, or immediately after. The symptoms generally commence abruptly. In the sopor of apoplexy, it is scarcely possible to rouse the patient to consciousness. Apoplexy, although

† Traité des Maladies des Enfans, &c. 8vo. Paris, 1828.

it is sometimes rapidly fatal, for instance within an hour, very often lasts a whole day, or even longer.

*b.* Epilepsy, distinguished by convulsions and abolition of sense, is generally a chronic disease, and has sometimes precursory symptoms. The fit begins violently and abruptly. The patient cannot, in general, be roused by external stimuli. In fatal epilepsy, the paroxysm generally lasts long, sometimes more than a day. Epilepsy is scarcely ever fatal in the first paroxysm.

*c.* The diseases of the spinal cord which, producing convulsions, delirium, and coma, may be confounded with narcotic poisoning, are extravasation of blood into the spinal cord; inflammation of the membranes; and inflammation and ramollissement of the cord itself. These diseases are not very probable sources of fallacy, but they serve to show the necessity of examining the spinal cord and its membranes in all judicial cases of alleged narcotic poisoning, especially where death has not been rapid. On comparing the effects of poisons with these diseases, we observe, generally, that poisoning with the narcotics has not of course any precursory symptoms, except by fortuitous concurrence. It has happened most frequently amongst the young, especially of the female sex; and the person may not have been corpulent or disposed to the diseases above alluded to. The effects of the common narcotics, when they prove fatal, begin not later than an hour, or at the utmost two hours, after they are taken: most frequently they begin in fifteen or thirty minutes. Hence, if it can be proved that the nervous symptoms under which a person died did not begin till several hours after he took food, drink, or medicine, it appears almost, if not absolutely, certain that a narcotic poison cannot have been the cause of death. There are some exceptions to this, but the rule will hold generally. With respect to the commonest of the narcotic poisons, opium, the symptoms never occur till after the interval of ten, twenty, or thirty minutes; and the deleterious gases, and hydrocyanic acid and its compounds, are the only poisons which act more instantaneously. Except with these latter, the sopor from poisoning is at first imperfect, and increases gradually, though sometimes very rapidly. In the sopor of apoplexy, it is possible to rouse the patient to consciousness; but, on the other hand, with some narcotics, and particularly with opium, the person may be roused from the deepest lethargy, if he be spoken to in a loud voice, or forcibly shaken, or if water be injected into the ear. Few people die of pure narcotic poisoning who outlive twelve hours, and the greater number die much sooner, in eight or six hours. On the other hand, the narcotic poisons rarely prove so rapidly fatal as apoplexy sometimes does. Apoplexy decidedly may occasion death in considerably less than an hour. The narcotic gases and prussic acid are the only narcotics which prove so rapidly fatal. The shortest known period in which death has been occasioned by opium is three hours.

In relation to epilepsy, it may be observed that, if we except some cases of poisoning with hydrocyanic acid and the narcotic gases, the effects of narcotic poisons are gradual, although their progress toward their extreme of violence is often rapid. In abrupt cases of poisoning with hydrocyanic acid, the poison, under certain conditions, will be found in the body; while, in sudden poisoning with the narcotic gases, the nature of the accident is rendered obvious to a cautious inquirer by the collateral circumstances. The variety of poisoning with which epilepsy is most apt to be confounded, that is, with hydrocyanic acid, has hitherto always proved fatal within half an hour after the symptoms begin, unless the dose has been small, and given repeatedly.

3. Of the symptoms of the narcotico-acrid class, as compared with the signs of natural disease.

The poisons of this class are all derived from the vegetable kingdom. They have a double action; the one local and irritating, the other remote, and consisting of an impression on the nervous system. In large doses, their narcotic effects are most conspicuous; in small doses, their irritant action. Their most conspicuous effect is injury of the nervous functions, and the symptoms are so analogous to those from narcotic poisons as not to require any distinct specification. They seldom prove fatal if the case last above twelve hours, that is, by their narcotic action. The poisonous fungi and digitalis are, however, exceptions to this remark.

### II. *Of the Evidence from morbid Appearances.*

The appearances after death which are really morbid, and which may be produced by poisons, are, from one great class, the signs of inflammation of the alimentary canal in its progressive stages; in another class, the signs of congestion within the head; and in the third, a combination of the effects of the two foregoing classes. But these appearances are not invariably occasioned by the poisons which usually cause them, and most of them are exactly similar to those left by many natural diseases. In general, therefore, the morbid appearances alone can never distinguish death by poison from the effects of natural disease.

Unusual lividity and early putrefaction were formerly much relied on as proofs of poisoning, but they are alike unfounded, and do not even justify suspicion. Arsenic, indeed, appears to have the power of preventing putrefaction under certain circumstances. In connexion with the symptoms and the general evidence, the appearance after death, by pointing out the nature of the previous illness, may furnish decisive evidence when the moral proof is strong; and, again, in cases of alleged *imputation of poisoning*, they are necessary to determine whether a poison actually found in the body was introduced during life or after death.* And, in cases where no doubt can be entertained that poison was taken, the evidence from morbid appearance may be necessary or useful for settling whether or not it was the cause of death. When signs of the action of poison are not found in the dead body, and, on the contrary, the effects of the operation of natural disease are discovered, the presumption is that the person died a natural death. But even here caution is necessary, and a positive opinion, in the absence of a *history of the symptoms*, occasionally impossible, for poisons sometimes leave no signs of their action; and, on the other hand, a pathologic state, which in one person has caused death, may have existed in another without producing fatal or very evident consequences. An inspector should not, therefore, so frame his reports as to exclude the possibility of a different cause from the apparent one, unless the appearances are such as must have been necessarily a cause of death.

1. Of the morbid appearances caused by the irritant poisons, compared with those of certain natural diseases.

The effects of the irritant poisons on the alimentary canal are those of inflammation and its consequences, from the slightest to the severest degree, the gullet participating occasionally in its severest consequences. Simple redness of the stomach, &c. is liable to the greatest doubts, and should never be regarded as an effect of inflammation, in the absence of some more advanced or decisive proof of inflammatory action. The most instructive information on this head has been furnished by Dr. Yellolyt and M. Billard.‡ Dr. Christison has pointed out an appearance of

* Orfila, Toxicologie générale, ii. 681.

† London Med. Chir. Trans. iv. 371.

‡ De la Membrane Muqueuse Gastro-intestinale, 1825.

the mucous membrane of the stomach, resembling melanosis, as an indication of inflammation. We must refer to his work (p. 104) for the description, which, we presume, will need further illustration. We have already said that Dr. Christison's inquiries render it probable that idiopathic *acute* gastritis is a very uncommon disease in this country. Reticulated lymph, adhering to the villous coat, and accompanied with corresponding reticulated redness of that coat, such as is seen in animals poisoned with arsenic or oxalic acid, is an unequivocal sign of inflammation. (P. 106.)

Ulceration and perforation are alike signs of natural disease and of poisoning. The co-existence of scirrhus furnishes a ground of distinction. The perforation from simple gelatinization of the coats, without proper inflammatory action, is the most puzzling and remarkable variety of perforation. Its most frequent situation is in the posterior surface of the stomach. It is sometimes small, and appears as if it had been made with a punch, but may be so large as to involve an entire half of the stomach, and sometimes there is more than one aperture. The margin is of all shapes, frequently fringed, and almost always formed of the peritoneum; the other coats being more extensively dissolved. But sometimes the peritoneal tunic of the stomach is most dissolved, intimating perhaps that, in these instances, the softening commences on the exterior coat. The organs in contact with the hole are frequently excavated or perforated. The pulp never smells of gangrene, nor does the edge of the whole in the stomach ever adhere to the adjoining organ.

The circumstances under which this extraordinary appearance occurs, are singularly various. Prof. Chaussier, and other French pathologists, conceive it to be always a morbid process; and without doubt it frequently is so. But it has been often discovered where the cause of death was quite obvious, and no suspicion could be entertained of disease of the stomach;* as, for example, in sudden deaths from convulsions after delivery; after death from suppura-

* Dr. Ebermaier, who has recorded several very interesting cases of perforation of the stomach, states that "in no instance was the nature of the malady suspected by the physicians. In some the symptoms were so obscure, that an affection of the stomach was never thought of. The fatal termination of the disease was never anticipated. Death sometimes occurred unexpectedly, almost in the midst of apparent health." (London Med. and Phys. Journ. 1828, pp. 302, 422.) Upon this important subject, much interesting information will be found in the Dict. des Sc. Med. t. xlvi. p. 314, art. *Perforation*, illustrated by plates.

tion of the brain, the result of violence; or in sudden death from fracture of the skull; or from hanging, and where no previous sign, referrible to disease of the stomach, had existed. The opinion of Hunter, supported by Allan Burns, remains unrefuted, we think, by the observations of the French pathologists.*

Now, in perforations produced by the irritant poisons, the margin is commonly of a peculiar colour: for example, yellow from nitric acid, brown from sulphuric or the muriatic acids, orange from iodine. But, says Dr. Christison, an infallible criterion, and one of universal application, is the following: Either the person dies very soon after the poison is introduced, in which case vital action may not be excited in the stomach; or he lives long enough for the ordinary consequences of violent irritation to ensue. In the former case, part of the poison will be found in the stomach; in the latter, the deep vascularity, or black extravasation, around the hole, and in other parts of the stomach, will at once distinguish the appearance from a spontaneous perforation. Spontaneous erosion is very generally combined with unusual whiteness of the stomach, and there is never any material vascularity. (See trial of Angus for the murder of Margaret Burns, 1808, for an illustrative example.)

2. On the morbid appearances left in the body by the narcotic poisons, contrasted with the effects of natural disease.

The morbid appearances which the narcotic poisons leave in the dead body are commonly insignificant, and, slight as they are, are not by any means invariably found. Congestion, extravasation, and the simple apoplexy of Dr. Abercrombie,† are alike the consequences of natural disease and narcotic poisoning; for, with respect to the latter, Dr. Christison says, "it might even be a fair subject of inquiry whether death from some narcotic poisons at least, such as opium, is any thing else than death from simple apoplexy." (P. 499.) Opium, however, has produced ex-

* Mr. Paxton, however, observes, "We have been in the habit of examining a great number of animals at different periods after death, and most of them carnivorous, whose gastric secretion is more active than that of the human stomach in dissolving animal matter; yet in these we never could find any erosion of the coats of the stomach, which must have been the case if it were possible for the gastric juice to have such effects. We consider the stomach, therefore, to be equally insensible to its presence in life or in death." (Paley's Natural Theology, illustrated by Plates and explanatory Notes, by James Paxton.)

† See p. 216 of his work on the Brain, &c. second edition.

travasation of blood; and congestion is not an unusual consequence of poisoning by it. (P. 541.) There is reason to believe that, in some cases of poisoning by opium, putrefaction takes place speedily.

After poisoning by hydrocyanic acid, the eye is said to retain a glistening and staring appearance, as during life. Such has been observed in several instances; and such is also the case after death from carbonic acid gas. Dr. Christison remarked the same fact very distinctly six hours after death, in a woman who died of cholera; and it has been noticed in cases of death taking place during the epileptic paroxysm.

The odour of prussic acid is oftentimes exhaled by the blood, but not always; and it is sometimes perceptible in the blood, even when it is not so in the stomach. Schubarth states as the result of his researches (Christison, 568,) that, if the dose is sufficient to cause death within ten minutes, the peculiar odour will always be remarked in the blood of the heart, lungs, and great vessels, provided the body have not been exposed to rain or to a current of air, and the examination be made within a moderate interval, twenty-four hours, for instance: but if the dose has been so small that life is prolonged for fifteen, twenty-seven, or thirty-two minutes, then, even immediately after death, it may be impossible to remark any of the peculiar odour, evidently because the acid is rapidly discharged by the lungs: and, even when the dose is large enough to cause death in four minutes, the smell may not be perceived, if the body have been left in a spacious apartment for two days, or exposed to rain for a few hours only.

### III. *On the Evidence of Poisoning from chemical Analysis.*

The chemical evidence, in charges of poisoning, is generally, and with justice, considered as the most decisive of all the branches of proof. It is accounted most valid when it detects the poison in the stomach, intestines, or gullet; then in the matter vomited; next in articles of food, drink, or medicine, of which the sufferer has partaken; and, lastly, in any articles found in the prisoner's possession, and for which he cannot account satisfactorily.

In two circumstances, however, some corroboration is necessary. In *imputation of poisoning*, it should be determined, by an accurate account of the symptoms, or by the morbid appearances, whether poison was introduced into the body before or after death;* and, granting that it was

* Vide Orfila, Toxicologie générale, ii. 681.

taken during life, whether it was the cause of death. Dr. Christison quotes the two following cases from German works, in illustration of the necessity of the latter inquiry.

A girl was severely chastised by her father, and died whilst the chastisement was being inflicted, and, as was supposed by the father and others, from its effects. The bruises were severe, but it appeared to Wildberg, inadequate to cause death. He therefore examined the cavities, and found the stomach very much inflamed, and lined with a white powder, which proved to be arsenic. It turned out that, on the theft, which the girl had committed, being detected, she swallowed arsenic, for fear of her father's anger; that she vomited during the flogging, and died in slight convulsions.

The other case occurred to Pyl, in 1783. A woman at Berlin, who lived on bad terms with her husband, went to bed in perfect health, but soon afterwards her mother found her breathing very hard, and discovered a wound on the left side of the breast. A surgeon being immediately sent for, the hemorrhage, which had never been great, was checked without difficulty; but she died towards morning. On opening the chest, the wound was found to penetrate the pericardium, but did not reach the heart; and, although the fifth intercostal artery had been divided, scarcely any blood was effused into the chest. Coupling these circumstances with the trifling hemorrhage during life, and the fact that she had much vomiting and some convulsions immediately before death, Pyl satisfied himself that she had not died of the wound; and the signs of corrosion in the mouth and throat, and of irritation of the stomach, with the discovery of the remains of some nitric acid in a glass in her room, proved that she had died of poison. (P. 49.)

But, if poison be not detected in the body, the experimenter being skilful, and the poison of a kind easily to be discovered, still it must not be concluded, from that fact alone, that poison has not been the cause of death; for it may have been all discharged by vomiting and purging, or it may have been all absorbed or decomposed.

1. It may have been discharged by vomiting or purging. The case of a grocer is quoted in the New York Med. and Phil. Journal, vol. iii., who died eight hours after swallowing an ounce of arsenic, and in whose body none could be found by chemical analysis. It is singular, however, how ineffectual vomiting sometimes proves in expelling some poisons from the stomach; for, after two days incessant vomiting, grains of arsenic have been found in the gullet of a person who survived the taking of the poison four days although none could be found in other parts.

2. The poison may have disappeared because it has been absorbed. In a case in which the moral circumstances left no doubt that laudanum had been swallowed seven or eight hours before death, none could be detected by Dr. Christison; and M. Desruelles has related the instance of a soldier, who died in six hours and a half after swallowing two drachms of solid opium, and in whose stomach nothing was found but a yellowish fluid, quite destitute of the smell of the drug.

3. Poisons may not be found because the excess has been decomposed. This is particularly the case with vegetable and animal poisons, which may be altogether destroyed by the process of digestion. Some mineral poisons, such as corrosive sublimate, lunar caustic, hydrochlorate of tin, are also decomposed in the stomach; but they are not removed beyond the reach of chemical analysis, for the basis of the poison may be found in the solid contents of the stomach, under some other compound form.

The decay of the body may render it impossible to detect some poisons; but, on the other hand, it appears that arsenic, under certain circumstances, has a preservative power. That poison has been detected fourteen months after interment.* (P. 261.)

IV. *Evidence of Poisoning from Experiments on Animals.*

Evidence from experiments on animals with articles supposed to contain poison, is more equivocal than was once imagined. The matter subjected to trial may be either suspected food, drink, or medicine, or it may be the stuff vomited during life, or found in the stomach after death.

1. The evidence derived from the effects of the suspected food, drink, or medicine, is better than that drawn from the effects of the vomited matter, or the contents of the stomach. But it should not be forgotten that what is poison to man is not always poison to the lower animals, and that, on the other hand, some of the lower animals are poisoned by substances not hurtful to man. According to the experience of Orfila, the cat and dog, but particularly the latter, are affected by almost all poisons exactly as man is. Alcohol, however, acts more powerfully on them than on man. It has been fully ascertained that arsenic, copper,

* A case has recently been published in La Lancette Francaise, which was communicated to the Académie Royale de Médecine by M. Orfila, in which arsenic was detected in a body *seven years* after burial.

mercury, the mineral acids, opium, strychnia, veratrum album, prussic acid, cyanogen gas, sulphuretted hydrogen, and many others, produce nearly the same effects on man, quadrupeds, birds, amphibious animals, and even on fishes and on insects. Hence there are cases in which the evidence from experiments on animals with suspected articles of food, is unequivocal. In the case of Mary Bateman, who, after cheating a poor family for a series of years, at last tried to avoid detection by poisoning them, it was justly accounted good evidence that a portion of the pudding and the honey supposed to have been poisoned caused violent vomiting in a cat, killed three fowls, and proved fatal to a dog in four days, under symptoms of irritation of the stomach, such as were observed in the people who died. But in such a case a moderately skilful chemist could scarcely fail to detect the poison.

2. In the case of the matters vomited, or the contents of the stomach, there are weightier objections to experiments on animals: for the poison which has caused death may have been partly or wholly vomited beforehand, or absorbed, or transmitted into the intestines, or decomposed by the process of digestion; or, though abounding in the matters vomited, or in that which remains in the stomach, it may be so much diluted as not to have any effect on an animal; or the animal fluids secreted during disease are believed to act occasionally as poisons.

The last objection is a very important one, but, in Dr. Christison's opinion, it has been a good deal exaggerated. He refers to the repeated and fatal experience of anatomists, together with the precise experiments of MM. Gaspard and Magendie for proofs of the poisonous effects of the animal fluids under disease; and he quotes also the isolated case related by Morgagni, of a child who died of tertian ague and in the midst of convulsions, in whose stomach was found an æruginous bile so deleterious that a little of it given with bread to a cock caused convulsions and death in a few minutes, and a scalpel stained with it, when thrust into the flesh of two pigeons, killed them in the same manner. On the whole it appears that, in the present state of our knowledge, experiments or accidental observations on the effects of the contents of the stomach or matters vomited on animals are equivocal in their import. At the same time it must be observed, that the effects of some poisons on man may be developed so characteristically on animals by the contents of the stomach, as to supply very pointed evidence: for instance, in the case of a girl who was proved to have died of accidental poisoning with laudanum, the inspector evaporated the contents of the stomach to dryness, made an alcoholic extract from the residue, and giving this to several dogs, chickens, and frogs, found that they were all made lethargic by it, and that a few died comatose.

### V. *On the moral Evidence in Cases of Poisoning.*

The observations of Dr. Christison on the moral proofs of poisoning are novel and important, but we have not space even to abridge them, and must content ourselves with a mere enumeration of the chief circumstances of which he takes cognizance.

1. Suspicious conduct on the part of the prisoner before the event; such as dabbling with poisons, when he has nothing to do with them in the way of his profession; conversing about them, or otherwise showing a knowledge of their properties not usual in his sphere of life.

2. The purchase or possession of poison recently before the date of the alleged crime, and the procuring under false pretences, such as for poisoning rats, when there are none on his premises to poison, or for purposes to which it is never applied.

3. The administration of poison, either in food, drink, or medicine, or otherwise.

4. The intent of the prisoner; such as the impossibility of his having administered the poison ignorantly, or by accident, or for beneficial purposes alleged or not alleged.

5. The fact of other members of the family besides the deceased having been similarly and simultaneously affected.

6. Suspicious conduct on the part of the prisoner during the illness of the person poisoned; such as directly or indirectly preventing medical assistance being procured, or the relations of the dying person being sent for, or showing an over-anxiety not to leave him alone with any other person, or attempting to remove or destroy articles of food or drink, or vomited matter which may have contained poison, or expressing a foreknowledge of the probability of speedy death.

7. Suspicious conduct after the person's death; such as hastening the funeral, preventing or impeding the inspection of the body, giving a false account of the previous illness, showing an acquaintance with the real or supposed effects of poison in the dead body.

8. The personal circumstances and state of mind of the deceased, his deathbed declaration, and other particulars, especially such as tend to prove the impossibility or improbability of suicide.

9. The existence of a motive or inducement on the part of the prisoner; such as his having a personal quarrel with the deceased, or a hatred of him, his succeeding to property by his death, or being relieved of a burden by it; his knowing that the deceased was with child by him, &c. (P. 61.)

We have thus presented to our readers an outline of the most important of the *general* subjects discussed in the work of Professor Christison. The opinion delivered by him, in opposition to almost all continental authorities, that the symptoms alone, in certain cases of poisoning, are capable of supplying decisive proof of the fact, is very important, and we think well sustained by some parts of the preceding abstract. The French, Germans, and others, have regarded this species of proof too much like men of abstract science, and with too little of common sense: for, granting that certain effects known to be the common results of certain poisons may, by remote possibility, be occasioned by natural disease, yet, if men engaged in extensive practice, and after the most diligent literary research, cannot, from their own experience, affirm, neither ascertain, that such effects have ever happened in more than one or two *doubtful* instances, this remote possibility ought surely, in strict propriety, to have but the very slightest influence over our conclusions. We are aware that solemn consequences attend our decisions, and hence, in forming them, extremely cautious investigation and unbiassed judgment should ever be exercised; but, under their guidance, if, in certain cases, we did not ascribe certain symptoms to the agency of poison, we must depart from all our usual methods of reasoning.

We designed to select a few of the subsequent chapters for abstract and remark, in order to afford our readers some notions of the manner in which Dr. Christison has treated of the individual poisons; but our limits forbid, at present, at least, the execution of our intention: and this we scarcely regret, as any condensed view offered by us could not supersede the advantage, and indeed necessity, of consulting the original work. The *treatment* pointed out in the various chapters on the individual poisons, renders the work valuable to the general practitioner; while to the medical jurist, from the novelty, accuracy, and practical bearing of the facts so copiously collected, it is indispensable. In short, it is, beyond comparison, the most valuable practical treatise on Toxicology extant.

*Researches principally relative to the Morbid and Curative Effects of Loss of Blood.* By MARSHALL HALL, M.D. F.R.S.E. &c. &c.—8vo. pp. 303. Seely and Burnside, London, 1830.

OF the many contributions to medical science for which we are indebted to Dr. MARSHALL HALL, none is more important than that which relates to the subject of the volume before us. In 1820 he published, in his Medical Essays, a brief sketch of some effects of loss of blood, and on exhaustion and sinking from various causes. Since that period his zeal has not abated, and he now submits to the profession a more elaborate and perfect exposition of those views and doctrines which he had before but briefly described.

The principal object of the present work is to apprise the inexperienced of some unexpected phenomena arising from loss of blood, of the remarkable difference in the degree of tolerance or intolerance of loss of blood in different diseases, of the equal danger of an inefficient and undue use of the lancet, and of a rule which may be adopted to obviate this danger. Another object has been to establish, "beyond the reach of controversy," a distinction between two classes of morbid affections, that of inflammations and that of irritations. Dr. Hall has very judiciously illustrated the general principles he has laid down by cases furnished by friends, and by the testimony of various highly respectable authors.

The work is divided into two parts; the first treating of the *morbid*, the second of the *curative* effects of loss of blood. The morbid effects of loss of blood are further divided into the *more immediate*, including syncope, convulsion, delirium, coma, sudden dissolution; and the *more remote* or exhaustive, with excessive reaction, with defective reaction, sinking, delirium, coma, and lastly with amaurosis. The effects of further loss of blood in cases of exhaustion, are next stated. They consist in the substitution of syncope for reaction, or in sinking or more sudden dissolution. The influence of various circumstances on the effects of loss of blood, as the strength, age, constitution, &c. of the patient, but especially of various diseases, is briefly commented upon in the fifth chapter. The chief effects of loss of blood on the *internal organs* are effusion and congestion in the brain, œdema of the lungs, effusion into the serous and cellular membranes, a deranged and tympanitic state of the alimentary canal. The last chapter of the first part contains the *treatment* of the effects of loss of blood.

It will be apparent from this rapid enumeration of the subjects of the first part of the work, that a new, interesting, and to a great extent unexplored, field of investigation is entered into. That Dr. Hall has very skilfully laboured in it, will be evident to our readers from the extracts we shall give. By due attention to the following very important statement, many fatal mistakes in practice may be avoided. We could cite many cases which have fallen under our own observation, in which the lancet has been employed to overcome the very symptoms the previously too free use of the lancet had occasioned.

"Some of the more obvious and striking effects of loss of blood, or those of reaction, are such as to suggest the idea of increased power and energy of the system, and of increased action in some of its organs, and to lead to an erroneous and dangerous employment or repetition of the lancet, when a directly opposite mode of treatment is required; while the state of actual but protracted sinking frequently resembles a state of oppression of the brain, or of congestion of the lungs, so accurately, as to prompt the unwary practitioner to a still more suddenly fatal use of the lancet.

"The result of this treatment is in itself again apt further to mislead us; for all the previous symptoms are promptly and completely relieved; and this relief, in its turn, again suggests the renewed use of the lancet. In this manner the last bloodletting may prove suddenly and unexpectedly fatal." (P. 5, 6.)

Proceeding in the order we have mentioned, Dr. Hall first describes the state of syncope through its more transitory, its more fearful, and its fatal forms. Convulsions are next treated of. From the phenomena attending the occurrence of convulsions, it is clearly denoted that the brain may be *similarly* affected in *opposite* states of the general system. Dr. Hall regards convulsion, when it arises from bloodletting, as a proof that the remedy has been carried too far. Delirium occurs as an *immediate*, as mania occurs as a more *remote*, effect of loss of blood. Several instances of the occurrence of delirium from exhaustion are detailed. Coma sometimes occurs as an immediate, but more frequently as a more remote, effect of loss of blood. Nothing can afford a more striking example of the necessity for the salutary warning which the author has given against mistaking cases of *reaction* for those of real power, than an instance he relates of the fatal effects of bloodletting during the former state. It should be read and re-read by the student, until every circumstance of it is indelibly imprinted upon his memory.

We proceed to the more remote effects of loss of blood, and first of exhaustion with *reaction*. The recovery from ordinary syncope is generally a simple return to a healthy state of the functions, or nearly so. After profuse loss of blood, the recovery is not quite so uniform; but in case the person be subjected to repeated bloodlettings, or to a continued drain,

"The pulse, instead of being slow and feeble, acquires a morbid frequency and a throbbing beat, and there are, in some instances, all the symptoms of excessive reaction.

"This state of excessive reaction is formed gradually, and consists, at first, in forcible beating of the pulse, of the carotids, and of the heart, accompanied by a sense of throbbing in the head, of palpitation of the heart, and eventually, perhaps, of beating or throbbing in the scrobiculus cordis, and in the course of the aorta. This state of reaction is augmented occasionally by a turbulent dream, mental agitation, or bodily exertion; at other times it is modified by a temporary faintness or syncope. There is also sometimes irregularity of the beat of the heart and of the pulse.

"In the more exquisite cases of excessive reaction, the symptoms are still more strongly marked, and demand a fuller description.

"The beating of the temples is at length accompanied by a throbbing pain of the head, and the energies and sensibilities of the brain are morbidly augmented; sometimes there is intolerance of light, but still more frequently intolerance of noise and of disturbance of any kind, requiring stillness to be strictly enjoined, the knockers to be tied, and straw to be strewed along the pavement; the sleep is agitated and disturbed by fearful dreams, and the patient is liable to awake or be awoke in a state of great hurry of mind, sometimes almost approaching to delirium; sometimes there is slight delirium, and occasionally even continued delirium; more frequently there are great noises in the head, as of singing, of crackers, of a storm, or of a cataract; in some instances there are flashes of light; sometimes there is a sense of great pressure or tightness in one part or round the head, as if the skull were pressed by an iron nail or bound by an iron hoop.

"The action of the heart and arteries is morbidly increased, and there are great palpitation, and visible throbbings of the carotids, and sometimes even of the abdominal aorta, augmented to a still greater degree by every cause of hurry of mind or exertion of the body, by sudden noises or hurried dreams or wakings. The patient is often greatly alarmed, and impressed with the feeling of approaching dissolution; the state of palpitation and throbbing are apt to be changed, at different times, to a feeling of syncope; the effect of sleep is in some instances very extraordinary, sometimes palpitation, at other times a degree of syncope, or an overwhelming feeling of dissolution; the pulse varies from 100 to 120

or 130, and is attended with a forcible jerk or bounding of the artery.

"The respiration is apt to be frequent and hurried, and attended with alternate panting and sighing; the movement of expiration is sometimes obviously and singularly blended with a movement communicated by the beat of the heart; the patient requires the smelling bottle, the fan, and the fresh air.

"The skin is sometimes hot; and there are frequently general hurry and restlessness.

"In this state of exhaustion, sudden dissolution has sometimes been the immediate consequence of muscular effort on the part of the patient, or of his being too suddenly raised from the recumbent into the erect position." (P. 29.)

It will be seen that the term "exhaustion with sinking" is not used to denote a state of negative weakness, but of positive and progressive failure of the vital powers. This state is altogether peculiar, and we believe it remained for Dr. Hall first to describe it. M. Andral has noticed it in his recent work on Pathological Anatomy, and compares it, as Dr. Hall did many years ago, to the condition of an animal whose pneumo-gastric nerves had been divided.

"The symptoms of exhaustion with excessive reaction may gradually subside, and leave the patient feeble, but with returning health; or they may yield to the state of sinking. This term is adopted not to express a state of negative weakness merely, which may continue long and issue in eventual recovery, but to denote a state of positive and progressive failure of the vital powers, attended by its peculiar effects, and by a set of phenomena very different from those of exhaustion with reaction.

"If in the latter the energies of the system were augmented, in the former the functions of the brain, the lungs, and the heart, are singularly impaired. The sensibilities of the brain subside, and the patient is no longer affected by noises as before; there is, on the contrary, a tendency to dozing, and gradually some of those effects on the muscular system which denote a diminished sensibility of the brain supervene, as snoring, stertor, blowing up of the cheeks in breathing, &c. Instead of the hurry and alarm on awaking, as observed in the case of excessive reaction, the patient in the state of sinking requires a moment to recollect himself, and recover his consciousness; is perhaps affected with slight delirium, and he is apt to forget the circumstances of his situation, and, inattentive to the objects around him, to fall again into a state of dozing.

"Not less remarkable is the effect of the state of exhaustion with sinking on the function of the lungs: indeed, the very first indication of this state is, I believe, to be found in the supervention of a crepitus in the respiration, only to be heard at first on the most attentive listening; this crepitus gradually becomes more audible and passes into slight ruttling, heard in the situation of the bronchia and trachea; there is also a degree of labour or oppression, sighing, hurry, blowing in the breathing, inducing acuteness in the nostrils, which are dilated below and drawn in above the lobes at each inspiration; in some cases there is, besides, a peculiar catching, laryngeal cough, which is especially apt to come on during sleep, and awakes or imperfectly awakes the patient.

"The heart has, at the same time, lost its violent beat and palpitation, and the pulse and arteries their bounding or throbbing.

"The stomach and bowels become disordered and flatulent, and tympanitic, and the command over the sphincters is impaired.

"The last stage of sinking is denoted by a pale and sunk countenance, inquietude, jactitation, delirium, and coldness of the extremities." (P. 44.)

We next come to the subject of exhaustion with *delirium*. This Dr. Hall regards as constituting a peculiar and not unfrequent form of mania, whether connected or unconnected with the puerperal state. Dr. Hall has perhaps some reason to complain that neither Dr. Abercrombie nor Dr. Gooch has referred distinctly enough to his important and original views on this subject.*

In the fourth chapter, some very important observations are made "on the effects of further loss of blood in cases of exhaustion." In confirmation of his views upon this subject, Dr. Hall refers to a recent communication of Mr. Brodie to the Medico-Chirurgical Society.

The treatment of the effects of loss of blood is very succinctly but clearly described.

"The constitutional treatment must be stimulant in syncope, sedative and soothing in the state of reaction, and restorative in that of sinking. The local treatment must vary with the organ chiefly affected, and with the mode in which it is affected.

"When syncope assumes a dangerous form, the principal remedies are, an attention to the posture of the patient, stimulants, and chiefly brandy, and the transfusion of blood.

"The effect of posture is not, even now, fully known. It would be easy to allow the patient to lie over the edge of the head, the head low upon the floor, and the feet greatly raised. In this manner such pressure would be restored to the encephalon as would in many cases support life until, other remedies being administered, the patient might be placed out of immediate danger.

"I need not, in this place, notice the importance of a regulated mode of giving brandy and nourishment. I think it is frequently

* Commentaries on some of the more important Diseases of Females. By Marshall Hall, M.D. &c. 1827.

given in such quantities as actually to induce sickness and its own rejection from the stomach, and so as to frustrate the object of the physician completely. The effect should be carefully watched. The physician ought not, of course, in such a case, to leave the patient for a moment.

"The next remedy is transfusion. Unfortunately it has too frequently happened that the proper period of adopting this measure has been allowed to pass by. Not only the vascular system is exhausted, but after a time the functions of the nervous system have begun to fail. It might be a question, therefore, whether galvanism might not be usefully conjoined with transfusion.

"It is an important point to determine how large a quantity of blood the system will bear to receive under various circumstances of exhaustion. Too much may overwhelm; too little may be inadequate to the accomplishment of the object in view.

"It is also an important question whether the operation should be done at once, or at twice or thrice, and with what intervals. As the system cannot bear a sudden reduction of the quantity of blood, so it may not be enabled to bear its too sudden restoration.

"It is almost needless to add, that a due attention must be constantly paid to assist the arterialization of the blood, by the admission of fresh air; and to sustain the animal heat by proper clothing, and especially warm applications to the feet.

"If there should be convulsions, delirium, or coma, it may be necessary to apply a sinapism to the nape of the neck; and in the two former cases some mild sedative, as the tinctura hyoscyami, may be of advantage.

"In the case of excessive reaction, the remedies appear to be, first, extreme quiet of body and of the mind; then the mildest sedatives, especially the hyosciamus; thirdly, the mildest nutriments; and lastly, and above all, time.

"The pain and throbbings in the head, the intolerance of noises, the general susceptibility to disturbance, the palpitations of the heart, alike demand the utmost quiet, to which every thing soothing in the manner and treatment must be added. The tinctura hyosciami is, I think, the kindliest anodyne and sedative in these cases. The cause, and other circumstances of the case, point out the necessity for mild nutriment, to which, perhaps, the minutest quantities of brandy may be added.

"It may be necessary to subdue the throbbing action of the head, by local bloodletting even; and it is most remarkable how small a quantity of blood being taken will relieve. An interesting example of this kind is given at page 95. Two or three leeches are frequently quite sufficient.

"But the most unequivocal remedy is a cold spirituous lotion applied all over the head, by means of a cap consisting of one fold of stocking.

"In exhaustion with delirium, the tinctura hyoscyami should be conjoined, in full doses, with the other remedies. The morbid susceptibility, not only of the brain, but of the heart, is greatly assuaged by this remedy.

"In cases of exhaustion with sinking, stimulants must be administered abundantly. Cataplasms of mustard may be applied to the nape of the neck and to the feet. It is difficult to imagine what would be the effect of the transfusion of blood; I have no doubt that galvanism would prolong life; and I think the two remedies might be conjoined with advantage.

"In all cases of exhaustion the functions of the bowels suffer. Constipation and flatulency are the usual consequences. These are best relieved by the warm water enema; which must, however, of course, be administered with due precaution, to prevent further exhaustion.

"It is interesting to observe the blunted sensibilities in syncope and in sinking, and to compare them with the morbidly acute sensibilities of the state of reaction. Sinapisms to rouse, and the tinctura hyoscyami to lull them, are, in their respective places, remedies of the greatest value.

"Sinapisms may tend to save or prolong life, in the sinking state, on the principle of exciting inflammation. For it will be seen shortly that, during a state of inflammation, the system is far less susceptible of the effects of loss of blood generally than in health." (P. 108.)

In several appendices, Dr. Hall treats of the similarity between the effects of loss of blood and the state of bloodlessness which exists in chlorosis; of a hydrencephaloid affection of infants arising from exhaustion, of which we recently gave a very full account; of exhaustion arising from abstinence, and of the sinking state in general.

In our next Number we shall give the substance of the second part of this very interesting work, which we cannot too strongly recommend to the notice of our readers. p. 226.

